# Severity of illness influences the microcirculatory response to red blood cell transfusion in the critically ill: an observational cohort study

**DOI:** 10.1186/s13054-020-03202-z

**Published:** 2020-08-12

**Authors:** Lisa van Manen, Jessica M. Deurvorst, Maike E. van Hezel, Margit Boshuizen, Robin van Bruggen, Nicole P. Juffermans

**Affiliations:** 1 Department of Intensive Care Medicine and Laboratory of Experimental Intensive Care and Anesthesiology, Amsterdam UMC, location AMC, University of Amsterdam, Meibergdreef 9, 1105 AZ Amsterdam, The Netherlands; 2 Department of Blood Cell Research, Sanquin research and Landsteiner Laboratory, University of Amsterdam, Amsterdam, The Netherlands; 3 Department of Intensive Care Medicine, OLVG Hospital, Amsterdam, The Netherlands

**Keywords:** Red blood cell transfusion, Anemia, Microcirculation, SOFA score

## Main text

Critically ill patients frequently require red blood cell (RBC) transfusions. The aim of giving a RBC transfusion is to increase oxygen delivery to tissues in anemic patients. The oxygen delivering capacity depends not only on the hemoglobin (Hb) level and oxygen saturation, but also on tissue perfusion. In current clinical practice, transfusion decisions are based on the Hb level only. However, baseline Hb level does not correlate with a positive effect of a RBC transfusion on tissue perfusion [1]. The effect of a RBC transfusion seems to depend on the baseline microcirculatory flow [1, 2]. However, routine measurements of the microcirculatory flow in clinical practice are difficult, since microcirculatory flow evaluation requires experience and the analysis of the videos is time-consuming. We hypothesized that organ injury level of the patient influences the microcirculatory response of the recipient to RBC transfusion, rendering the Sequential Organ Failure Assessment (SOFA) score an easy bedside value which could aid in transfusion decision-making.

We performed sublingual Sidestream DarkField (SDF) imaging (Microscan, MicroVision Medical, Amsterdam, The Netherlands) within 1 h before and after transfusion of 1 leucodepleted RBC unit in 18 anemic non-bleeding critically ill patients on a tertiary intensive care. The study was ethically approved by the medical ethical committee of the Amsterdam University Medical Centre (NTR 6596; NL61833.018.17). Written informed consent was obtained from all participants or their legal representatives. Software AVA 3.2, MicroVision Medical, Amsterdam, The Netherlands, was used to analyze the videos and to calculate the “De Backer” score (including vessel density, proportion of perfused vessels (PPV) and perfused vessel density (PVD)) and the Microvascular Flow Index (MFI) [3]. Wilcoxon signed-rank test was used to compare paired data. Correlation coefficient between the delta of the SDF values (difference between before and after transfusion) and SOFA score was calculated using the Spearman correlation. *p* values of the correlations are corrected for multiple testing with Holm-Bonferroni correction. A *p* value of less than 0.05 was considered statistically significant. Statistical analyses were performed using SPSS ((IBM SPSS Statistics, version 25).

Patient characteristics are shown in Table 1. Median Hb level at baseline was 6.8 (IQR 6.4–7.3) g/dL and did not correlate with baseline microcirculatory flow (PPV: rho = − 0.141, *p* = 0.567, PVD: rho = − 0.445, *p* = 0.192, MFI: rho = − 0.276, *p* = 0.534). In line with previous findings, the effect of a transfusion depended on the baseline microcirculatory flow, with patients with impaired baseline values showing an improvement and patients with normal baseline values showing a deterioration (PPV: rho = − 0.772, *p* = 0.000, PVD: rho = − 0.795, *p* = 0.000, MFI: rho = − 0.697, *p* = 0.001).

**Table 1:** Patient characteristics. Data are median with IQR or number with percentage

	All patients (*n* = 18)
Gender, male	10 (56%)
Age (years)	61 [57–66]
SOFA score	7 [6–11]
Specialism, surgical	10 (56%)
Sepsis	7 (39%)
Mean arterial pressure (mmHg)	76 [72–82]
Baseline hemoglobin level (g/dL)	6.7 [6.4–7.2]
Vasopressive medication	6 (33.3%)
Lactate (mmol/L)	1.7 [1.2–1.8]
pH	7.45 [7.42–7.49]
Arterial saturation	94.2 [93.2–94.8]
Storage duration RBC unit, days	16 [11–21]
Hospital mortality	4 [22.2%]

**Fig. 1:**
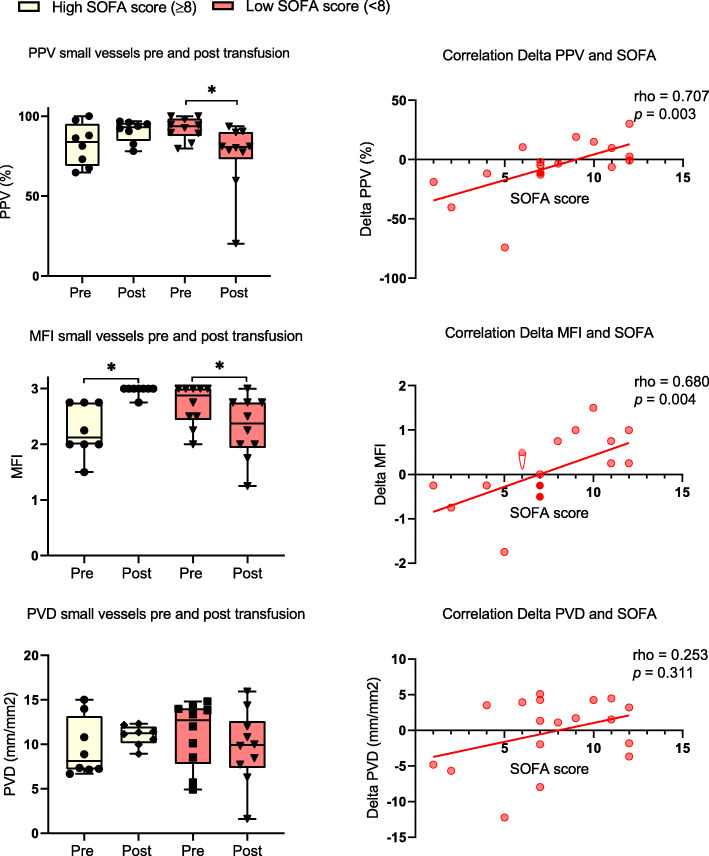
SDF variables before and after transfusion and correlations between Delta SDF (small vessels) and SOFA score. Pre, before transfusion; post, after transfusion; PPV, proportion of perfused vessels; MFI, microvascular flow index; PVD, perfused vessel density. Rho is Spearman’s rank correlation coefficient. *p ≤ 0.05

No correlation was found between baseline SDF values and the SOFA score (PPV: rho = − 0.382, *p* = 0.288; PVD: rho = − 0.089, *p* = 0.726; MFI: rho = − 0.404, *p* = 0.288) indicating that the severity of illness does not correspond with the flow in the microcirculation before transfusion. However, there was a positive correlation between SOFA score and the change in PPV and MFI values after transfusion, in which the microcirculation improved in the patients with a SOFA score > 8 but deteriorated in those with lower SOFA scores. PVD did not correlate with the SOFA score (Fig. 1).

In conclusion, these results suggest that patients with high SOFA scores have improvement of their microcirculation after a RBC transfusion, while patients with a low SOFA score show a decrease in flow and perfusion following a transfusion. This suggests that we should take the severity of the illness of the patient into account when deciding whom to transfuse, when microcirculatory flow analysis is not available.

## Data Availability

The datasets used and/or analyzed during the current study are available from the corresponding author on reasonable request.

## Abbreviations

RBC: Red blood cell
Hb: Hemoglobin
SOFA: Sequential organ failure assessment
SDF: Sidestream DarkField imaging
PPV: Proportion of perfused vessels
PVD: Perfused vessel density
MFI: Microvascular Flow Index
